# Compression shorts reduce prenatal pelvic and low back pain: a prospective quasi-experimental controlled study

**DOI:** 10.7717/peerj.7080

**Published:** 2019-06-20

**Authors:** Jaclyn M. Szkwara, Wayne Hing, Rodney Pope, Evelyne Rathbone

**Affiliations:** 1 Department of Physiotherapy/Faculty of Health Sciences and Medicine, Bond University, Robina, QLD, Australia; 2 School of Community Health, Charles Sturt University, Thurgoona, NSW, Australia; 3 Faculty of Health Sciences and Medicine, Bond University, Robina, QLD, Australia

**Keywords:** Pregnancy, Low back pain, Pelvic pain, Compression garments, Dynamic elastomeric fabric orthoses (DEFOs), Maternity pelvic support, Prenatal care, Physiotherapy

## Abstract

**Background:**

Common prenatal ailments negatively impact performance of activities of daily living and it has been proposed that the use of dynamic elastomeric fabric orthoses, more commonly referred to as compression garments, during pregnancy might aid in the reduction of pain from these ailments, allowing for improved functional capacity. However, the effectiveness of such garments in this context has not been established. This study aims to determine whether compression shorts are effective and thermally safe in the prevention and management of prenatal pelvic and low back pain (LBP).

**Method:**

A prospective quasi-experimental controlled study using parallel groups without random allocation was conducted, involving 55 childbearing women (gestational weeks 16–31) recruited from hospital and community-based maternity care providers. The compression shorts group (SG) wore SRC Pregnancy Shorts in addition to receiving usual care. The comparison group (CG) received usual care alone. Primary outcome measures—Numeric Pain Rating Scale (NPRS) and Roland Morris Disability Questionnaire (RMDQ) and secondary measures Pelvic Floor Impact Questionnaire - 7 (PFIQ-7) and SF-36 Short Form Health Survey—were assessed fortnightly over 6-weeks for both groups. The compression SG self-assessed daily their body temperatures to monitor thermal impact. Data analysis involved descriptive analyses of the primary and secondary outcome measures scores by group and time-point, and multivariable linear regressions to assess between-group differences in change scores at 6-weeks from baseline while controlling for baseline factors.

**Results:**

After controlling for baseline scores, gestational weeks and parity, statistically significant differences in NPRS and RMDQ change scores between groups were in favour of the compression SG. At 6-weeks, mean (SD) NPRS change scores in the compression SG and CG were significantly different, at −0.38 (2.21) and 2.82 (2.68), respectively, *p* = 0.003. Mean (SD) RMDQ change scores in the compression SG and CG were also significantly different, at 0.46 (3.05) and 3.64 (3.32), respectively, *p* = 0.009. A total of 883 (99.7%) of the reported daily self-assessed body temperatures ranged between 35.4 and 38.0 °C when wearing the compression shorts. At 6-weeks, mean (SD) PFIQ-7 and SF-36 change scores in the compression SG and CG were not significantly different.

**Conclusion:**

Compression shorts are effective and thermally safe for prenatal management of pelvic and LBP.

**Registration:**

Trial registration was not required (Australian Government Department of Health Therapeutic Goods Administration (TGA), 2018).

## Introduction

The two most commonly reported prenatal problems are low back pain (LBP) and pelvic girdle pain (PGP) (Fitzgerald & Mallinson, 2012; Pennick & Liddle, 2013). An estimated 50–70% of women report LBP during pregnancy (Colla, Paiva & Thomas, 2017; Hughes et al., 2018; Ho et al., 2009a; Wang et al., 2005). On this basis, LBP is quite often considered ‘normal’ during pregnancy, but women report that it interferes with their activities of daily living (Colla, Paiva & Thomas, 2017; Hughes et al., 2018). PGP during pregnancy is estimated to range in prevalence from 24% to 50%, with the cited reason for this range being there is variability in how PGP is defined, how physical assessments to identify PGP are performed and how it is reported (Clinton et al., 2017; Fitzgerald & Mallinson, 2012; Walters, West & Nippita, 2018). Research suggests the two common prenatal ailments negatively impact activities of daily living (Clinton et al., 2017) and, in Scandinavian countries, they have been found to account for a large proportion of sick-leave taken by pregnant women or postnatal women returning to work (Backhausen et al., 2018; Aldabe, Milosavljevic & Bussey, 2012; Larsen et al., 1999; Stuge, Hilde & Vøllestad, 2003).

Abnormal pelvic floor muscle function can affect the timing of voluntary contraction and relaxation of these muscles and occurs in women whose primary complaints are LBP or PGP (Fitzgerald & Mallinson, 2012). For this reason, many current treatments for prenatal pelvic pain incorporate interventions to address these abnormalities in muscle function (Fitzgerald & Mallinson, 2012). Multimodal interventions incorporating, for example, physiotherapy, pelvic support belt (Hammer et al., 2015) and/or complementary medicine (described in the literature as massage therapy, acupuncture, relaxation, yoga and hypnosis (Waterfield et al., 2015; Field, 2008)) have been found to be effective in relieving prenatal LBP and PGP (George et al., 2013; Ho et al., 2009b; Pennick & Liddle, 2013; Walters, West & Nippita, 2018).

Pelvic support belts constitute a type of intervention used to provide relief from several common complaints during pregnancy. Research has found 83% of women with PGP or LBP experience reduced posterior pelvic pain when wearing a pelvic support belt, with only 12% experiencing no relief, and 5% reporting they felt worse (Depledge et al., 2005). Furthermore, training of the diagonal trunk muscle system, (specifically internal and external oblique muscles, latissimus dorsi, multifidus and gluteus maximus), and the use of pelvic support belts together assist women to achieve greater reductions in symptoms than are typically achieved by spontaneous resolution alone (Mens, Snijders & Stam, 2000; Stuge, Holm & Vøllestad, 2006). Pelvic support belts provide often-needed support to reduce prenatal pain and so such belts tend to be an early intervention provided to manage pain in this context (Bertuit et al., 2018; Carr, 2003; Clinton et al., 2017; Vleeming et al., 2008). However, other factors need to be considered when prescribing pelvic support belts, given the target population is pregnant women. These factors include levels of compliance in wearing the belt, positioning of the belt, accuracy in sizing, required compression location to stimulate stabilizing muscles, and effects on core body temperature (a special consideration, as this needs to be carefully regulated during pregnancy) (Damen et al., 2002; Ho et al., 2009b).

Earlier studies indicated that pelvic support belts were associated with poor compliance due to ‘garment-related problems including skin irritation from the seams and fasteners, discomfort from rolled up and buckled back panel during sitting and unsatisfactory adjustability, fitting for back support and the noise of Velcro tape’ (Ho et al., 2009a). Therefore, a valuable direction for future research would be to investigate whether the use of compression garments might be superior to the use of pelvic support belts for therapeutic purposes in these populations, since research on compression garments has indicated an improved venous blood flow because of its compressive effects (Houghton, Dawson & Maloney, 2009); it can be worn under daily attire (Houghton, Dawson & Maloney, 2009); and its ability to decrease muscle oscillation which in turn may improve neurotransmission and mechanics (Doan et al., 2003).

Compression garments need to be clearly defined and described in research designed to test their effectiveness, feasibility and acceptability. Descriptions of specific garment types should identify the method of application of the garment, the garment design, the garment materials and the pressure applied by the garment (MacRae et al., 2012), so that study results can be validly interpreted, synthesised, compared and implemented. Review of the literature on compression garments is currently challenging, as it is difficult to compare and draw conclusions about the garments used in different studies because studies have used various garment types and application procedures (Ho et al., 2009a). The heterogeneity in designs and materials used in previous studies makes valid comparisons and synthesis of results from those studies difficult. For example, some studies have used standard compression bandages ranging in number of layers, others have used compression stockings or hosiery, and still other studies have used a sports compression garment. Furthermore, compression garments are variously defined and can be labelled using terms such as ‘maternity support garments’, ‘lumbopelvic support garments’, ‘pelvic stability’ or ‘support belts.’ A defined classification system for grading of the compression provided by garments is lacking, and design materials have been inconsistent. Therefore, the literature suggests the term dynamic elastomeric fabric orthoses (DEFOs) to be used when referring to compression garments as they are designed to apply consistent compression through tailored elastomeric panels strategically positioned to address common prenatal ailments (Sawle, Freeman & Marsden, 2016), minimising variability in garments used in this population.

The available research on DEFOs hints that use of DEFOs during pregnancy might aid in the reduction of pain associated with common musculoskeletal ailments, allowing for a potential increase in functional capacity. However, definitive evidence of such an effect is lacking. Consistent with the need for maternity care providers to utilise evidenced-based practice to provide effective and safe interventions for their patients, it is imperative that further research is conducted on DEFOs to assess their effectiveness in reducing pain and increasing functional capacity in pregnant women and to further confirm their thermal safety in this population, in order to guide their use in practice.

On this basis, the aims of this study were to examine the effectiveness of a specific DEFOs (SRC Pregnancy Shorts, manufactured by SRC Health Pty Ltd, Port Melbourne, Australia) for reducing prenatal pain and associated disability arising from the pelvic girdle and lower back and to further assess the thermal safety of these compression shorts when they were worn during pregnancy.

Based on published evidence, it was hypothesised that: (i) the use of DEFOs would be considered an effective therapeutic intervention to decrease prenatal PGP and LBP allowing an increase in functional capacity; (ii) perceived quality of life would improve with the use of DEFOs; and (iii) DEFOs will not affect maternal core temperature and therefore, would be thermally safe to wear during pregnancy.

## Method

### Ethics

Ethics approval for the study was granted by the Queensland Health Office of Human Research Ethics Committee (HREC/14/QGC/200) and Bond University Human Research Ethics Committee (BUHREC: RO1800c).

### Study design

A prospective quasi-experimental controlled study using parallel groups without random allocation was conducted and involved two groups: a compression shorts group (SG), which received usual physiotherapy and broader health care and wore SRC Pregnancy Shorts (SRC Health Pty Ltd, Melbourne, Australia), and a comparison group (CG), which received only usual physiotherapy and broader health care.

A randomised controlled (RCT) trial was initially proposed; however, following consideration of relevant ethical issues, consultation with the ethics committees and receipt of their considered recommendations, it was decided to allow participants the opportunity to decide which group they preferred to participate in. The key concern of the ethics committee was withholding of a possibly needed intervention from pregnant women. While a prospective quasi-experimental controlled study without random allocation is less rigorous than a randomised design, the use of a parallel CG meant that the study methodology was as rigorous as possible given the concerns of the ethics committee. As our study did not involve ‘unapproved’ therapeutic goods, it was not subject to registration as a clinical trial under either clinical trial notification scheme or clinical trial exemption according to the Australian Government Department of Health Therapeutic Goods Administration (TGA) (2018).

### Participant recruitment

Participant recruitment took place at a metropolitan hospital, local university and the facilities of maternity care providers on the Gold Coast, Australia. Recruitment occurred over a 2-year period, from March 2015 to March 2017. Recruitment strategies consisted of general information sheets posted in maternity care providers’ offices, information provided online and in newspaper articles, and referral from other participants involved in the study. Eligibility criteria for participation included: female, age 18–50 years, gestational weeks 16–31 at time of recruitment, diastasis of the rectus abdominis muscle (DRAM) less than six cm and complaining of LBP and/or PGP. Participants were not excluded if they were experiencing pelvic oedema, pelvic floor dysfunction, urinary or faecal incontinence, or mild varicose veins. A sample size calculation indicated that a total sample size of 47 was required for a multivariable linear regression with four predictors to detect a large effect size (Cohen’s *f*^2^ = 0.35) with a statistical power of 80% and significance level of 0.025 for the two primary outcomes. The sample size was inflated to 60 to allow for 20% dropouts.

Before commencement of recruitment for the study, four information sessions were given to the physiotherapists, nurses and midwifery staff at maternity care facilities that were involved in recruitment. These information sessions were used to explain the study and its procedures, and to discuss any questions or concerns about the research, the DEFOs, how to correctly fit the DEFOs, and how the recruitment, consent and enrolment processes were to be executed. Recruitment was conducted by the maternity care providers and once a person agreed to be involved in the study and with the prospective participant’s consent, their contact information was forwarded to the chief investigator. The chief investigator contacted the prospective participant, provided further information on the study and gained their voluntary, written informed consent if they agreed to participate. The participant then chose their involvement preference within the research study, in either the SG or CG. Subsequently, the participant was allocated a random number to use when accessing the survey (discussed further below). Participants were provided with the chief investigator’s contact details as part of the information and consent process, in case assistance was needed at any time.

### Intervention

The intervention for the SG group consisted of provision and wearing of SRC Pregnancy Shorts, together with their usual prenatal physiotherapy and broader health care. Participation in the study had no influence on their usual care. Participants in the SG were measured and fitted for the compression shorts and provided with one pair of compression shorts to wear daily for a minimum of 8 h per day during their pregnancy from date of recruitment for a total of 6-weeks. It was expected that they were to wash and dry the compression shorts as needed to enable the participant to wear them daily. Participants in the SG recorded daily their levels of compliance indicating the number of days and the average number of hours the SRC Pregnancy Shorts were worn daily over 2-weeks and reasons for any lack of compliance.

**Figure 1:**
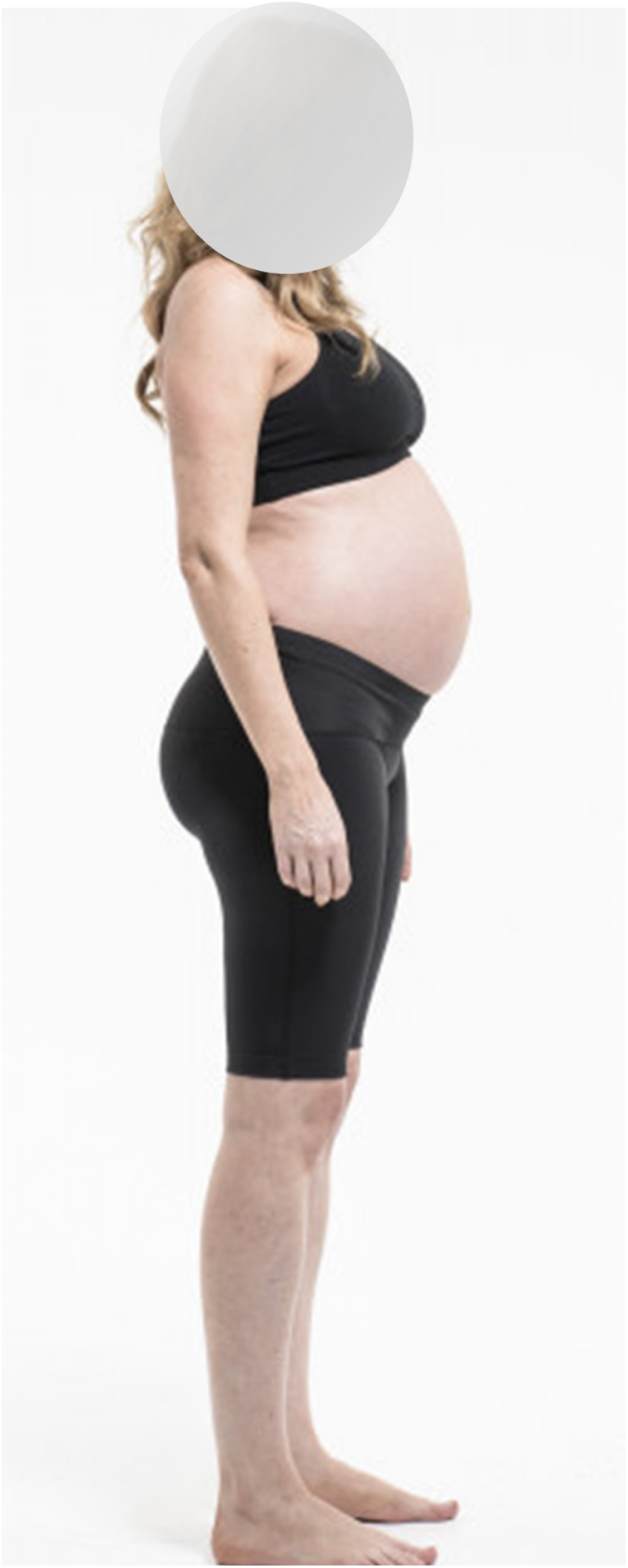
SRC Pregnancy Shorts—the DEFOs used by the SG. Available at https://www.srchealth.com/src-pregnancy-shorts-black.

SRC Pregnancy Shorts were specifically designed for pregnant women and are available in seven sizes, ranging from double extra-small to double extra-large. The SRC Pregnancy Shorts were constructed of a breathable warp knit fabric with a power mesh lining design using true cross compression so that no compression was lost during activity, as each fabric layer was cut on a different plane (SRC Health Pty Ltd, 2017) (Fig. 1).

The SG was instructed on how to wear the garment, how to adjust it for size as needed, and how to wash and care for the garment. More specifically, the SG participants were educated on wearing the garment so that the garment was firm around the pelvis, not tight or restrictive, and comfortable. Participants were instructed to use the adjustable tabs on each side of the garment, gradually releasing one side at a time until a comfortable fit was achieved.

The SG received a handout explaining precautions to take while wearing the garment (i.e. DEFOs to be firm and comfortable, not tight or restrictive and must sit below the belly), and any clinical presentations that required immediate cessation of use and to be reviewed immediately by their maternity-care provider (i.e. garment to be removed if body temperature was greater than 38 °C as this may indicate an illness). Whilst previous literature suggests that DEFOs do not increase core temperature, in order to monitor any possible risk, the SG received a handout explaining the precautions in detail. The participants wore the compression shorts daily for a minimum of 8 h per day unless clinical concerns (described above) indicated they should remove the garment and notify their maternity care provider.

The CG continued their usual prenatal physiotherapy care and broader health care, and participation in the study had no influence on their usual care. Participants in the CG did not receive a compression garment at any time. All participants were asked to notify the chief investigator on the research team should they be required to wear a DEFOs during their usual care, and if so, they would be excluded from the study.

For the SG and the CG, standard physiotherapy care was dependent on the individual needs of the participant and may include any of the following: joint mobilisations, massage therapy, exercise therapy, education, ergonomic and activities of daily living modifications, and general advice.

### Outcome measures

A baseline questionnaire completed online for the SG and CG included a brief demographic survey asking questions such as the group they were in, their age, gestational week, gravida/parity, and whether they had experienced DRAM (previous or current) or prenatal ailments or received treatment for either. SG and CG completed two primary outcome measures, the Numeric Pain Rating Scale (NPRS) (Huskissin, 1974) and the Roland Morris Disability Questionnaire (RMDQ) (Morris, 1983), and two secondary outcome measures, the Pelvic Floor Impact Questionnaire - 7 (PFIQ-7) (Barber, Walters & Bump, 2005), and the SF-36 Short Form Health Survey (Ware & Sherbourne, 1992; Marosszeky, 2014) each fortnight, online. The NPRS is an 11-item scale that subjectively measures the intensity of pain (Huskissin, 1974). Looking at aspects such as functional mobility, pain and activities of daily living, the RMDQ is a 24-item self-report questionnaire that assesses how LBP affects functional activities (Morris, 1983). The PFIQ-7 is a 7-question self-report questionnaire composed of three subscales that assess the impact of pelvic floor functioning or dysfunction on quality of life, daily activities and emotional health (Barber, Walters & Bump, 2005). The SF-36 short form health survey is a 36-item questionnaire that measures quality of life across eight domains that are physically and emotionally based (Ware & Sherbourne, 1992; Marosszeky, 2014). The SG group had an additional secondary outcome measure, daily self-assessed body temperatures.

### Data collection

Data were collected online via a link to the anonymous questionnaire at a time and place that was convenient for the participant. Responses for each participant in the SG and CG were linked across time-points (baseline, 2 weeks, 4 weeks and 6 weeks) using the participant’s random identification number. SG and CG participants completed the primary and secondary outcome measures, (NPRS, RMDQ, PFIQ-7 and SF-36), each fortnight, online. In addition, the SG self-assessed their body temperatures four times each day while wearing the compression shorts (at 2, 4, 6 and 8-h time points following donning of the compression shorts) and documented their daily average for their survey responses online for their body temperature. If the participant in the SG had to cease wearing the compression shorts at any time during any day due to their body temperature being greater than 38.0 °C, the highest body temperature was recorded for that day. The SG indicated in the online survey how their daily body temperature was measured when wearing the compression shorts, such as by measuring oral, axillary, tympanic, or rectal temperature.

### Data analysis

Data were analysed using IBM SPSS software (Version 24; Armonk, NY, USA). Descriptive statistics were reported as mean (SD) for normally distributed continuous variables and counts (%) for categorical variables. The assumption of normality for primary and secondary outcome measures was assessed by Q-Q plots, histograms and the Shapiro–Wilk test. Summary statistics were also produced for the different time-points (baseline, 2 weeks, 4 weeks, 6 weeks) for the raw primary and secondary outcome measures and for the change scores (from baseline) on these measures. Univariable regression analyses were conducted to assess the effects on primary and secondary outcome measures of factors including treatment group, baseline score, age category, gestational weeks and parity. Factors which were significant at the 0.10 level of significance and/or clinically important were selected for inclusion in subsequent multivariable analyses.

Multivariable linear regression analyses were used to examine effects at week 6 of group and other above-mentioned factors on the primary outcome measures, NPRS and RMDQ, and secondary outcome measures, PFIQ-7 and SF-36, in order to test the effects of wearing the SRC Pregnancy Shorts on a participant’s pain, quality of life and functional capacity while accounting for baseline score, gestational week and parity. Supplementary analyses included assessments of observed effects at weeks 2 and 4. The main results are presented as regression coefficients with 95% confidence intervals (CIs), and *p*-values, and the profiles of the mean change scores over time were graphically presented. The criterion for statistical significance in the regression analyses was set at the 0.025 level for the primary outcome measures and at the 0.05 level for the secondary outcome measures and supplementary analyses.

## Results

**Figure 2:**
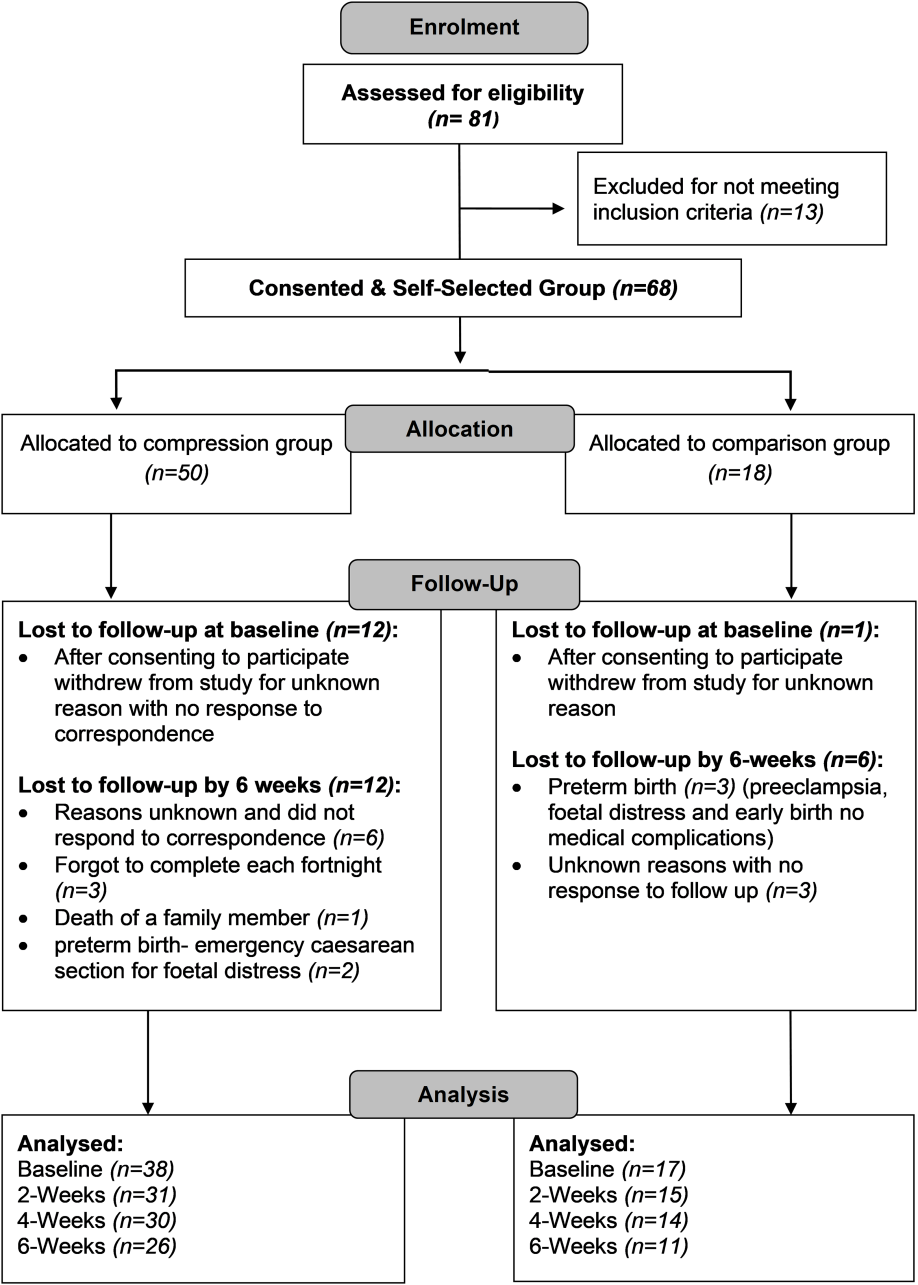
Participant flow diagram.

Of the 55 pregnant women who consented to participate and received their allocated intervention, 12 women were lost to follow-up in the SG and six in the CG by the final time-point (6 weeks) (Fig. 2). No participants were excluded from the CG based on the criteria that if a DEFOs was prescribed the participant would be excluded from the study. Table 1 describes the demographic characteristics of the participants, by group.

**Table 1:** Baseline demographic and clinical characteristics for the 55 pregnant participants.

Characteristics	Compression shorts group SG^a^ (*n* = 38)	Comparison group CG^b^ (*n* = 17)
Age category		
23–30	12 (31.5)	2 (11.8)
31–34	15 (39.5)	12 (70.5)
35–42	11 (28.9)	3 (17.6)
Gestational weeks mean (SD)	23.7 (4.9)	25.3 (4.0)
First pregnancy		
Yes	15 (39.5)	6 (35.3)
Gravida		
1	15 (39.5)	14 (82.3)
2	13 (34.2)	3 (17.6)
3	9 (23.7)	0 (0.0)
4	0 (0.0)	0 (0.0)
5	1 (2.6)	0 (0.0)
Parity		
0	15 (39.5)	6 (35.3)
1	16 (42.1)	10 (58.8)
2	7 (18.4)	0 (0.0)
3	0 (0.0)	0 (0.0)
4	0 (0.0)	1 (5.9)
Current DRAM^c^		
Yes	2 (5.3)	3 (17.6)
Current DRAM treatment		
Tubing band	0 (0.0)	2 (11.8)
Pilates	0 (0.0)	1 (5.9)
Previous DRAM		
Yes	28 (73.7)	12 (70.6)
Previous DRAM treatment		
Physiotherapy	5 (13.2)	1 (5.9)
Tubing band	2 (5.3)	2 (11.8)
HEP^d^	6 (15.8)	1 (5.9)
Pilates	2 (5.3)	1 (5.9)
Acupuncture	0 (0.0)	0 (0.0)
Surgery	0 (0.0)	0 (0.0)
None	3 (7.9)	1 (5.9)

**Notes:**

Data are counts (*n*(%)) unless otherwise specified.

a SG wore SRC pregnancy shorts (SRC Health Pty Ltd., Port Melbourne, Australia) and received usual care.

b CG received usual care only.

c Diastasis of the rectus abdominis muscle (DRAM).

d Home exercise program (HEP).

Participant demographic data were similar in the SG and CG when considering age category, whether it was the participant’s first pregnancy, gravida, parity and gestational weeks. DRAM only affected a very small number of participants in each group (SG (*n* = 2) and CG (*n* = 3)) and so was excluded as a predictor variable from the subsequent regression analyses. In the few participants who reported DRAM, differences were identified between the SG and CG in the size of DRAM at baseline (mean (SD) of 0.42 (1.81) and 2.67 (1.15) cm, in the SG and CG, respectively) and the types of treatment received for DRAM. Many women presented clinically at baseline with LBP (19 in the SG and 12 in the CG) and PGP (29 in the SG and 10 in the CG), as well as reporting other common pregnancy related ailments like urinary incontinence (Table 2).

**Table 2:** Frequencies (%) of participants with specific prenatal ailments at baseline.

Prenatal ailment	Compression shorts group—SG (*n* = 38)	Comparison group—CG (*n* = 17)
Low back pain	19 (50.0)	12 (31.6)
Pelvic girdle pain	29 (76.3)	10 (58.8)
DRAM	5 (13.2)	0 (0.0)
Vulval varicosities	13 (34.2)	1 (5.9)
Urinary incontinence	2 (5.3)	3 (17.6)
Muscle weakness	4 (10.5)	5 (29.4)
None	1 (2.6)	1 (5.9)
Other^a^	8 (21.1)	6 (35.3)

**Note:**

a Other included self-reported aching thighs, sciatic pain, leg varicose veins, thoracic and rib cage pain, hip pain, vaginal discharge, leg cramps and swelling of hands and feet.

Between time points (baseline to 2 weeks, 2–4 weeks and 4–6 weeks), the SG wore the compression shorts on average 10 h per day, meeting the compliance goal of a minimum of 8 h per day over a 14-day period. Those participants who did not wear the garments for a minimum of 8 h per day reported non-compliance reasons such as the garment being too hot or uncomfortable, and the participant not liking the appearance of the compression shorts when worn under work attire and summer dresses. The participants wore the compression shorts on average 10 out of 14 days, identifying reasons for non-compliance in days worn such as ‘forgot,’ ‘uncomfortable,’ ‘did not fit under work attire,’ and ‘the garment needed to be washed.’ Non-compliance in wearing the compression shorts increased as the women entered later weeks of their pregnancy. In the final 2-week time period, between weeks 4 and 6, SG participants wore the compression shorts for a mean of 10.2 h per day, on 9.4 days out of 14.

Assessment of the distributions of continuous variables indicated that the assumption of normality was met across all outcome measures. Table 3 provides raw data and change scores for the primary outcome measures, NPRS and RMDQ, and Table 4 displays raw data and change scores for the secondary outcome measures, PFIQ-7, SF36-PCS and SF36-MCS. Table 5 shows the estimated regression coefficients from the multivariable linear regression, to show the effects at week 6 of wearing the compression shorts on the changes in primary outcome measures, NPRS and RMDQ, after adjusting for baseline covariates.

**Table 3:** Raw data and change scores for NPRS and RMDQ.

Time	Raw data scores				Change scores^a^			
	*n*	SG Mean (SD)	*n*	CG Mean (SD)	*n*	SG Mean (SD)	*n*	CG Mean (SD)
NPRS^b^								
Baseline	38	4.21 (2.36)	17	2.94 (2.14)				
Week 2	31	3.03 (2.12)	15	3.27 (2.28)	31	−1.19 (1.89)	15	0.40 (1.35)
Week 4	30	3.33 (2.56)	14	4.86 (2.63)	30	−0.97 (2.31)	14	1.86 (2.11)
Week 6	26	3.81 (2.59)	11	6.09 (2.30)	26	−0.38 (2.21)	11	2.82 (2.68)
RMDQ^c^								
Baseline	38	4.05 (5.21)	17	4.18 (2.43)				
Week 2	31	2.97 (3.53)	15	5.00 (3.80)	31	−0.77 (4.80)	15	1.20 (2.91)
Week 4	30	3.87 (5.04)	14	6.21 (4.79)	30	0.03 (3.44)	14	2.37 (3.83)
Week 6	26	4.31 (6.27)	11	7.64 (3.85)	26	0.46 (3.05)	11	3.64 (3.32)

**Notes:**

a Negative change score indicates a reduction in pain or disability from baseline.

b Numeric Pain Rating Scale (NPRS) is scored 0 (no pain) to 10 (worst pain imaginable).

c Roland Morris Disability Questionnaire (RMDQ) is scored 0 (no disability) to 24 (maximum disability).

**Table 4:** Raw Data and Change Scores for PFIQ-7, SF36-PCS and SF36-MCS.

Time	Raw data scores				Change Scores^a^			
	*n*	SG Mean (SD)	*n*	CG Mean (SD)	*n*	SG Mean (SD)	*n*	CG Mean (SD)
PFIQ-7^b^								
Baseline	38	43.73 (30.34)	17	37.25 (47.21)				
Week 2	31	40.37 (31.16)	15	42.22 (48.82)	31	−1.25 (14.89)	15	3.81 (24.76)
Week 4	30	39.21 (39.72)	14	48.30 (52.46)	30	−3.65 (28.54)	14	7.14 (24.76)
Week 6	26	41.67 (39.00)	11	50.22 (51.79)	26	−0.46 (24.30)	11	2.17 (25.85)
SF36- PCS^c^								
Baseline	38	30.23 (4.17)	17	22.13 (6.44)				
Week 2	31	27.43 (5.35)	15	23.22 (6.23)	31	−2.31 (6.25)	15	1.87 (7.42)
Week 4	30	28.64 (4.65)	14	28.44 (3.41)	30	−0.52 (5.32)	14	7.55 (6.10)
Week 6	26	28.87 (6.27)	11	30.39 (4.41)	26	−0.23 (6.95)	11	9.64 (5.74)
SF36-MCS^d^								
Baseline	38	33.97 (6.32)	17	33.51 (6.53)				
Week 2	31	33.78 (5.73)	15	33.89 (7.65)	31	−0.37 (4.65)	15	0.09 (6.85)
Week 4	30	22.23 (5.62)	14	41.29 (9.78)	30	−12.14 (7.99)	14	7.76 (10.49)
Week 6	26	32.83 (6.06)	11	33.05 (8.59)	26	−1.27 (5.49)	11	0.62 (8.74)

**Notes:**

a A negative change score indicates a reduction in perceived effect of pelvic dysfunction on quality of life or an increase in level of functioning from baseline.

b Pelvic Floor Impact Questionnaire - 7 (PFIQ-7) is scored 0 (lowest perceived effect of pelvic floor dysfunction on quality of life) to 300 (greatest perceived impact of pelvic floor dysfunction on quality of life).

c SF-36 Physical Component Summary (SF-36 PCS) is the mean percentage score from all of the physically relevant questions (0% worst to 100% best possible level of functioning).

d SF-36 Mental Component Summary (SF-36 MCS) is the mean percentage score from all of the emotionally relevant questions (0% worst to 100% best possible level of functioning).

**Table 5:** Estimated regression coefficients from the multivariable linear regression to show the effect at week 6 of wearing compression shorts (*n* = 26) when compared to the control condition (*n* = 11) on the change in primary outcome measures, NPRS and RMDQ, after adjusting for baseline covariates.

Variable	Regression coefficient	95% Confidence interval	*p*-value
NPRS			
Constant	4.03	[−1.12–9.19]	0.121
Group^a^	−2.84	[−4.63 to −1.04]	0.003^c^
Baseline NPRS score	−0.41	[−0.79 to −0.04]	0.032
Gestational weeks^b^	0.003	[−0.18–0.19]	0.970
Previous number of children	0.09	[−1.01–1.18]	0.875
RMDQ			
Constant	4.09	[−3.14–11.32]	0.258
Group^a^	−3.39	[−5.89 to −0.89]	0.009^c^
Baseline RMDQ score	−0.001	[−0.24–0.24]	0.994
Gestational weeks^b^	−0.03	[−0.30–0.24]	0.523
Previous number of children	0.49	[−1.09–2.07]	0.531

**Notes:**

a Group was coded as 0 = CG and 1 = SG. A negative regression coefficient indicates a smaller change score for the SG, which indicates a more favourable result for the SG when compared to the CG.

b Recorded gestational weeks at baseline.

c Statistically significant at the 0.025 level.

**Figure 3:**
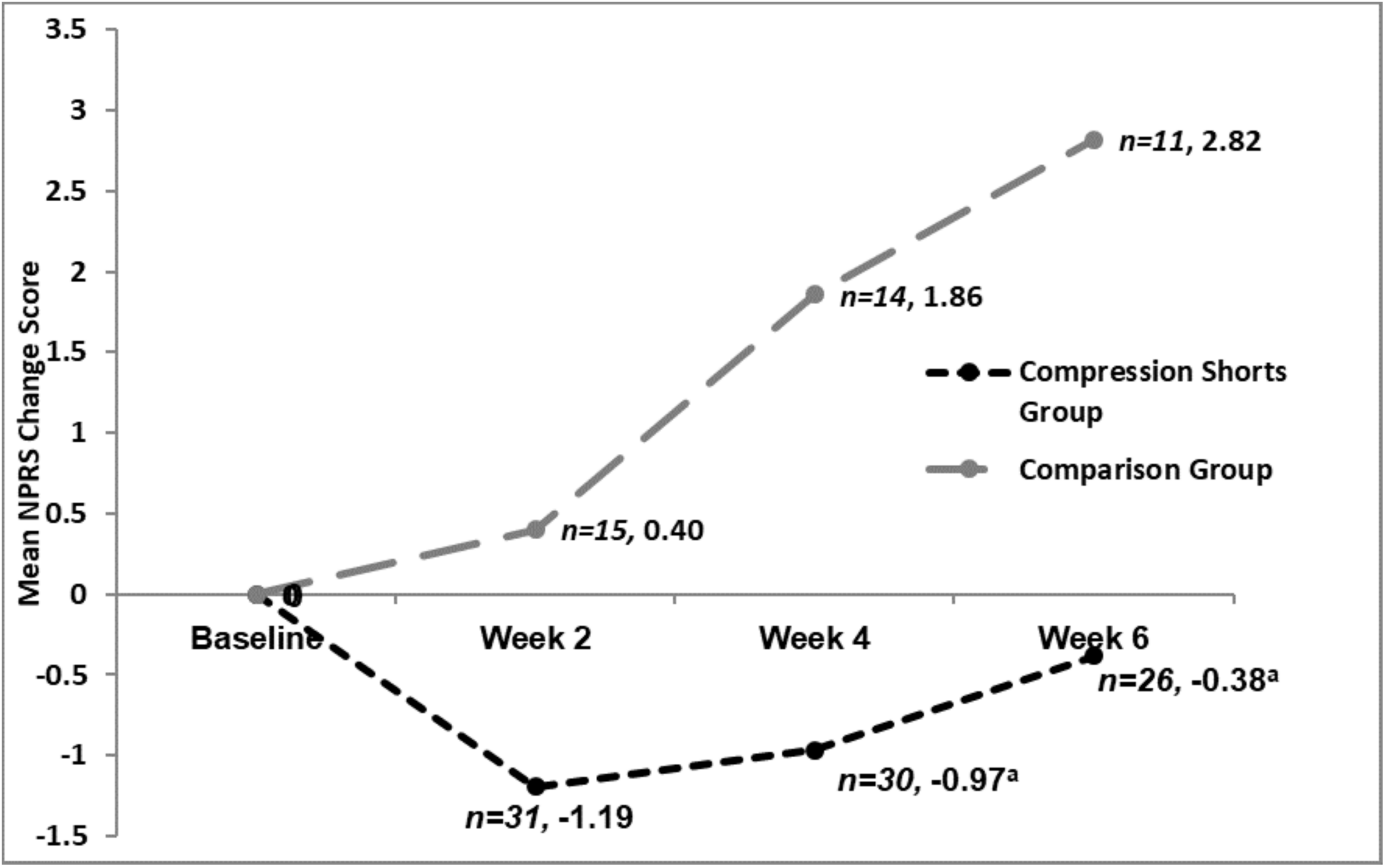
Mean NPRS change scores at each time point, by group. ^a^Statistically significant difference between groups. A negative change score indicates a reduction in pain from baseline.

The multivariable linear regression model detected statistically significant differences between the SG and CG in NPRS change scores at two of the time points, 4 weeks and 6 weeks, with the SG group reporting substantially better pain relief than the CG group. At 6 weeks, the SG group experienced a reduction in mean (SD) NPRS pain score (−0.38 (2.21)) from baseline levels, whereas the CG group suffered an increase in pain (2.82 (2.68)) over this same 6-week period (Table 3; Fig. 3). After controlling for baseline pain, age, gestational weeks and the number of previous births, 6 weeks after commencing use of the DEFOs there was a statistically significant difference in NPRS change scores between the groups (mean difference −2.84; (95% CI [−4.63 to −1.04], *R*^2^ = 0.387, *p* = 0.003), in favour of the SG group (Table 5).

**Figure 4:**
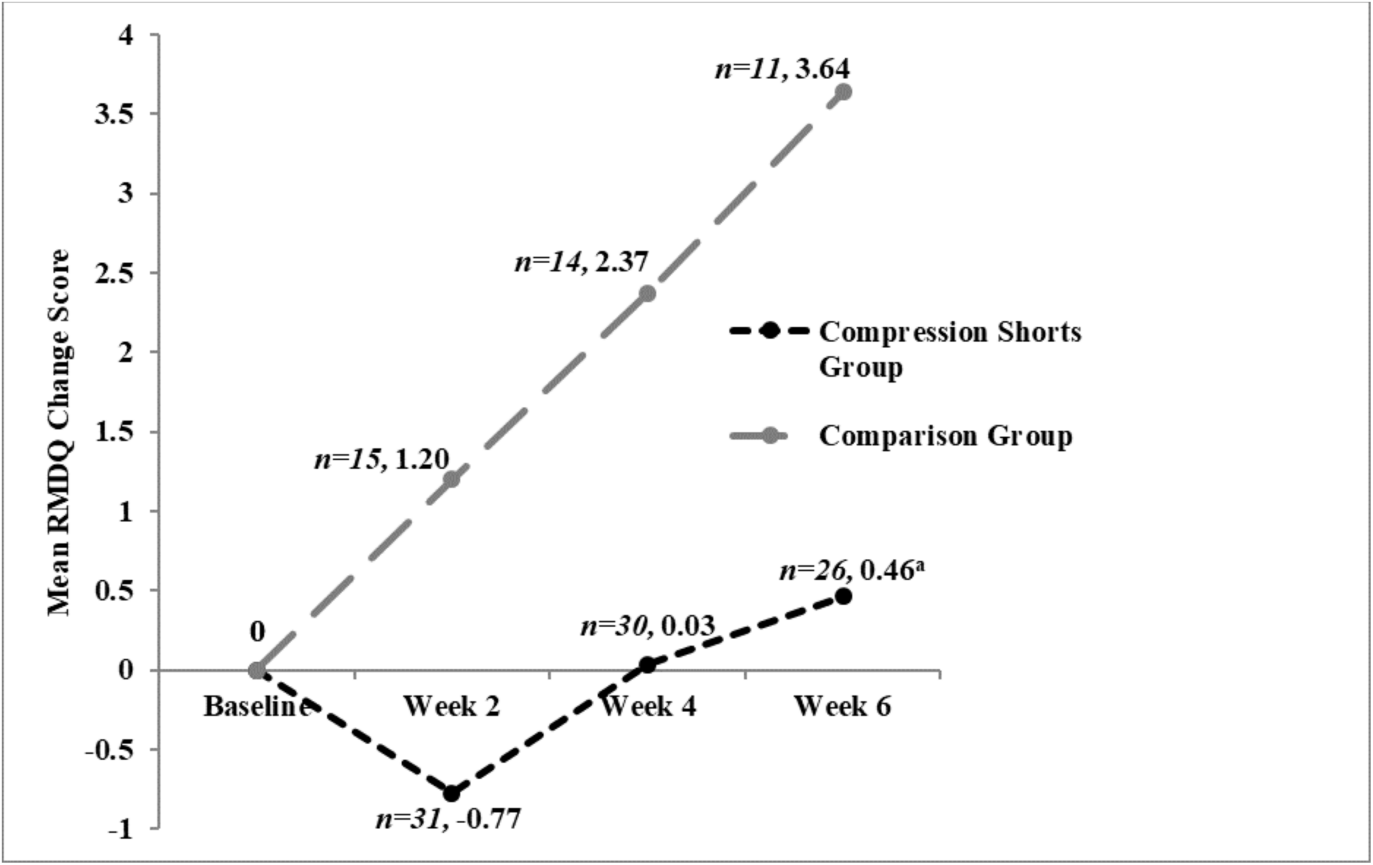
Mean RMDQ change scores at each time point, by group. ^a^Statistically significant difference between groups. A negative change score indicates a reduction in disability from baseline.

Differences between the groups in RMDQ change scores at 6 weeks reached statistical significance, with mean (SD) RMDQ change scores in SG and CG of 0.46 (3.05) and 3.64 (3.32), respectively, (Table 3; Fig. 4). These statistically significant differences were maintained after adjusting for baseline scores (pain or disability), gestational weeks and the number of previous births (mean difference: −3.39; (95% CI [−5.89 to −0.89], *R*^2^ = 0.198, *p* = 0.009), with the difference favouring the SG (Table 5).

There was no statistically significant difference between the SG and CG in PFIQ-7 change scores at 6 weeks (Table 4), when assessed using either unadjusted analyses via univariable linear regression or adjusted analyses with multivariable linear regression. PFIQ-7 total score ranges from 0 to 300 and to achieve a minimal clinically important difference (MCID) is a decrease of 36 points. PFIQ-7 raw scores at 6-weeks indicated that the SG attained a greater clinical improvement with MCID of 21.8 points compared to the CG with a MCID of 16.2 points. The SG reported a lesser negative effective with an average increase in score of 25.7 points compared to the CG reporting an average increase of 50.8 points. Although greater improvements were observed in the SG than the CG, neither group demonstrated a MCID for the PFIQ-7.

Change scores on the SF-36 Physical Component Summary (SF-36 PCS; Table 4) were also not significantly different between the groups at 6 weeks, either in the univariable analysis or in the multivariable analysis, which adjusted for baseline factors including baseline score on the SF-36 PCS, gestational weeks and number of previous births. The MCID for SF-36 PCS and SF-36 MCS is an increase of four points. Both groups achieved MCID for the SF-36 PCS. The SG demonstrated a MCID of 6.3 points and the CG a MCID of 9.4 points.

Group allocation was not a significant predictor of 6-week change scores on the SF-36 Mental Component Summary (SF-36 MCS; Table 4), in the univariable analysis or when adjustment was made for baseline scores on the SF-36 MCS, gestational weeks and number of previous births in the multivariable regression analyses. MCID was demonstrated in both groups, where the SG achieved a MCID of 7.5 points and the CG a MCID of 5.6 points.

Of the 885 reported daily self-assessed body temperatures in the SG, 883 (99.7%) ranged between 35.4 and 38.0 °C while participants were wearing the DEFOs; two (0.3%) self-assessed body temperatures were reported to be 39.0 °C. There were nil reports of any ailments for either report of body temperatures at 39.0 °C and cessation of wearing the SRC Pregnancy shorts occurred for one participant for 4 days and then returned to wearing the garment and monitored as per the research guidelines. The other occurred in the final week of study participation and therefore, did not return to wearing the garment as the study had been completed with nil reports of any ailments.

## Discussion

This prospective quasi-experimental controlled study evaluated for the first time the effectiveness of wearing custom designed compression shorts during pregnancy to manage prenatal PGP and LBP and associated disability and to further assess the thermal safety of these compression shorts when they were worn during pregnancy. The study responds to previous research which has highlighted that LBP and PGP are the most common conditions experienced by women during pregnancy (Colla, Paiva & Thomas, 2017; Hughes et al., 2018; Ho et al., 2009a; Wang et al., 2005). The primary aim of this study was to examine the effectiveness of a specific DEFOs for reducing prenatal pain and associated disability arising from the pelvic girdle and lower back and secondly this study aimed to further assess the thermal safety of DEFOs during pregnancy.

The main findings of significant differences between groups for the primary outcome measures, NPRS and RMDQ, indicated that wearing compression shorts during pregnancy resulted in reductions in pain and in disability related to LBP. After accounting for potential confounding variables, including the initial pain or disability score amongst other factors, there was a statistically significant difference between the groups in the change in level of pain and disability that occurred over the 6 weeks following commencement of participation and wearing of the compression shorts by the SG; the SG experienced substantially greater pain relief and less disability due to LBP than the CG. Therefore, the results of the NPRS and RMDQ scores for this study supported the first hypothesis that the use of DEFOs like the SRC Pregnancy Shorts are an effective therapeutic intervention for the management of pelvic pain and LBP and associated disability in pregnant women.

The management and reduction of pain results found in this study align themselves with some of the main findings from previous studies in the literature. Previously published literature suggests that using supportive/compressive belts and maternity garments can be effective in alleviating these conditions (Ho et al., 2009a; Wang et al., 2005; George et al., 2013; Pennick & Liddle, 2013; Depledge et al., 2005; Ramelet, 2002). Previous prenatal research has also indicated that DEFOs have a role in the reduction of back pain in particular instances such as sleeping, walking, working and getting up from a sitting position (Bertuit et al., 2018; Kalus, Kornman & Quinlivan, 2008).

Despite previous research, the use of individually-fitted compression shorts to reduce pain and improve functioning during pregnancy has not been previously studied. Compression shorts may be viewed more favourably than other types of compression belts and garments during pregnancy, for reasons. These include the minimal time required to appropriately fit the compression shorts, therefore not negatively impacting on maternity care provider’s current schedules, the garment being more tolerable and relatively comfortable to wear under daily attire, and the continued true cross compression (Doan et al., 2003). The SRC Pregnancy Shorts used in this study are practical and can be washed as needed to allow optimal results.

Although DEFOs demonstrated to be effective in the reduction of pain and disability, the results of this study did not show any significant differences between groups on the perceived impact on the participants’ quality of life based on the PFIQ-7 and SF-36 results. However, the lack of significant differences between groups may be explained by the Hawthorne effect which is where a participant may improve or alter an aspect of their behaviour or responses during research as a result of acute awareness of being studied (McCambridge, Witton & Elbourne, 2014). The PFIQ-7 and SF-36 scores were considerably low at both baseline and at 6 weeks in both the SG and the CG for our study therefore potentially demonstrating a participant bias of results. The PFIQ-7 and SF-36 findings in our study differ from the published literature by Adamczyk et al. (2013). Adamczyk et al. (2013) reported that based on the results of quality of life questionnaires and health surveys completed by participants, DEFOs are associated with a high level of treatment satisfaction and improve a participant’s quality of life. The study did indicate similar results to Adamczyk et al. (2013) when considering the clinical improvement of the participants as the PFIQ-7 and SF-36 MCS raw scores at 6 weeks indicated that participants that wore DEFOs attained a greater clinical improvement reporting a lesser negative effective on the perceived impact of quality of life than those that did not wear DEFOs. Although a strong conclusion based on the second hypothesis that perceived quality of life would improve with the use of DEFOs was not observed perhaps further research inclusive of greater numbers, random participant allocation, and controlling for factors such as participant bias with improved self-awareness may present stronger empirical evidence.

Recommendations in the prenatal literature as described by Brearley et al. (2015) indicate ‘that the safe upper limit for maternal core temperature is 38.9 or 1.5 °C above resting core temperature,’ to prevent possible harm to the foetus (Brearley et al., 2015; Hartgill, Bergersen & Pirhonen, 2011; Lindqvist et al., 2003). During this study, the participants remained below this threshold in 99.7% of instances (883 instances) where body temperature was measured while the compression shorts were worn, with only two (0.3%) reports of single measurements of body temperature reaching 39.0 °C which were monitored as per normal practice in prenatal care with their maternity care provider.

A concern for the use of compression garments during pregnancy is the increase in skin temperature and apprehension that it will raise core body temperature. According to 2016 guidelines from the Royal Australian and New Zealand College of Obstetricians and Gynaecologists Royal Australian and New Zealand College of Obstetricians and Gynaecologists (RANZCOG) (2016) a rise in core temperature of less than 1.5 °C is widely accepted as safe during pregnancy and these and other experts routinely advise pregnant women to avoid overheating to prevent possible harm to the foetus (Royal Australian and New Zealand College of Obstetricians and Gynaecologists (RANZCOG), 2016; Brearley et al., 2015; Lindqvist et al., 2003). Previous empirical evidence suggested male and female athletes, aged 19–33, that the thermoregulatory response to wearing compression garments at recreational and high levels of training and competition did result in increased skin temperatures (Doan et al., 2003); however, the associated increases in core temperature were not great enough to have detrimental effects in ambient and temperate environments with relative humidity between 46% and 64% (Houghton, Dawson & Maloney, 2009; MacRae et al., 2012).

Therefore, published literature suggests that there are several indications for the use of DEFOs during pregnancy as an intervention tool and concerns about the negative implications of increased core temperature for both the woman and foetus has proven to be safe in not raising core temperature based on high quality evidence available on the use of compression garments in other fields of health and medicine. Based on published evidence and the appropriate body temperature results of this study it can be suggested that DEFOs will not affect maternal core temperature and therefore, would be thermally safe to wear during pregnancy in support of the final hypothesis for this study.

### Limitations

The small CG size, the lack of randomisation in group allocation, and the relatively large number of participants lost to follow-up in both groups, mean that the results of this study should be considered with some caution as our findings may not have the generalizability to the wider population and warrant further research. As this study did not have sufficient numbers to be able to subdivide based on gestational weeks, future research with greater participant numbers may allow for sub analyses which may result in different clinical findings. This study did not include extensive qualitative data collection on DEFOs and therefore, future research examining the feasibility and acceptability of such garments is indicated. Nevertheless, the findings of the current study, as the first study to investigate the effectiveness and thermal safety of using compression shorts during pregnancy, are promising and indicate that compression shorts are thermally safe, with the study providing a stepping stone to larger and more rigorous, RCT studies, from an ethical viewpoint.

## Conclusion

This study demonstrated a reduction in pain and disability associated with common prenatal ailments when specially-designed and individually-fitted compression shorts were worn during pregnancy, indicating that these garments constitute a valuable and effective adjunct to usual healthcare for women during pregnancy. This study did not demonstrate an increase in the participant’s perception of their quality of life or mental health with use of the compression shorts, but it is possible any such effects were obscured in the current study by the relatively small sample sizes and losses to follow up in each group. Further research is therefore needed in these areas. Overall compliance for wearing the compression shorts during pregnancy was high, indicating women found the garments acceptable and reasonably comfortable overall. While wearing the compression shorts, the body temperatures of women remained within the acceptable range, indicating the compression shorts are thermally safe to wear during pregnancy. Although an improvement in quality of life was expected but did not occur, the results did indicate that the majority of women wearing the DEFOs did not experience a dramatic negative effect on quality of life and either improved slightly or stayed the same.

### Clinical implications

The study confirms that specially-designed and individually-fitted compression shorts like the SRC Pregnancy Shorts are an effective, thermally safe, non-pharmacological option for the prevention and management of low back and pelvic pain and associated disability during pregnancy. The results indicate their use is effective in decreasing numeric pain rating scores and reducing the effects of pregnancy related ailments such as LBP and PGP on the functional capacity and activities of daily living of women. The ease of wearing the garment, the ability to adjust the compression garment throughout the pregnancy and the breathable material make its use recommendable to pregnant women. Education to maternity care providers with respect to effective use of compression shorts for addressing common prenatal ailments and complaints may be valuable, as it could increase the awareness of maternity care providers regarding the effectiveness of these sorts of compression shorts in minimising the discomfort experienced by women during pregnancy.

### Implications for future research

Pregnancy related LBP and PGP are common ailments experienced by women during pregnancy and can have a negative impact on their functional capacity, activities of daily living and overall well-being. As this study was non-randomised and was limited by participant numbers and loss to follow up, further research is needed to confirm and extend the findings in this study so that maternity care providers can have an increased confidence in the prescription of compression garments.

## Additional Information and Declarations

### Data Availability

The following information was supplied regarding data availability: The raw data cannot be shared for publication with the article as informed consent was not received from the participants nor was approval obtained from the HREC. The data was made available for peer review.
